# Lectures on Diseases of the Skin

**Published:** 1887-01

**Authors:** Henry Wile

**Affiliations:** Lecturer on Dermatology, Atlanta, Ga.


### LECTURES ON DISEASES OF THE SKIN.

DELIVERED AT THE ATLANTA MEDICAL COLLEGE DURING THE SESSION OF 1885–86, BY HENRY WILE, M. D., LECTURER ON DERMATOLOGY, ATLANTA, GA.

### LECTURE XI.

ECZEMA — STAGES—VARIETIES, ERYTHEMATOSUM, PAPULOSUM, VESICULOSUM, PUSTULOSUM, RUBRUM, SQUAMOSUM, FISSUM—ACUTE—CHRONIC.

Reported Stenographically for THE ATLANTA MEDICAL AND SURGICAL JOURNAL by W. Kay Tewksbury.

ECZEMA (tetter) is an acute or chronic inflammatory process of great practical importance on account of its common occurrence, constituting, according to the best statistics, about thirty-three and one-third per cent. of the whole number of diseases of the skin. It presents a variety of symptoms and appears in a variety of forms, each of which were, until recently, regarded as distinct affections. It remained for Hebra to recognize the common pathology of these various forms and to group them together under one name. Notwithstanding this variety, he saw that certain features were always present which served to characterize the disease and make it a clinical unity. All the cutaneous manifestations may be divided into three stages: 1. Stage of congestion. 2. Stage of eruption. 3. Stage of desquamation. The disease is always ushered in by an active or passive congestion which, in typical cases, is followed by an eruption of papules, vesico-papules, vesicles or pustules that sooner or later disappears and ends in desquamation, followed by a gradual return to the normal condition. The stage of congestion may, however, terminate directly in desquamation without the intervening stage of eruption. The disease is divided into the following varieties, named according to the predominant symptom:

| Varieties | Stage |
|---|---|
| Eczema erythematosum. | 1. Stage of congestion. |
| Eczema papulosum.<br>Eczema vesiculosum.<br>Eczema pustulosum.<br>Eczema rubrum. | 2. Stage of eruption. |
| Eczema squamosum.<br>Eczema fissum. | 3. Stage of desquamation. |

These varieties result from a regular development of the disease as it passes through the above stages, and though apparently extensive, shows plainly a clinical unity. One variety may pass into another or several of them may be present in a single case. There may be combinations depending upon the cause, extent, localization and severity of the disease, also upon possible complications.

Itching or burning is a characteristic symptom and may be considered a *sine qua non*. It may be constant, but more often occurs in paroxysms. It varies in severity, being more distressing in some varieties than in others.

*Eczema erythematosum* appears in the form of variously-sized spots or diffuse areas of a bright red or violaceous color, which shade off gradually into the surrounding skin. It is usually localized and affects the face, especially the forehead and eyelids, the flexures of the large joints and those parts where surfaces are in opposition, as about the thighs. The skin becomes thickened and about the eyelids may even present an œdematous condition. It is accompanied by itching, more frequently a sensation of burning, pursues, as a rule, a chronic course and ends by passing into a stage of desquamation. This variety may be in combination with any of the others and may at any time readily pass into any of them.

*Eczema papulosum* consists of an eruption of pinhead-sized, pale or dark red discrete papules, at times coalescing to form large spots. Itching is a most distressing symptom. The patient soon scratches off the tops of the papules, and from slight hemorrhages these become capped with minute dark crusts. The lesions may run their course as papules or develop into vesicles, resulting in a mixture of lesions, the variety being then called *vesico-papular eczema.* Papular eczema attacks especially the flexor surfaces and may be localized or generalized.

*Eczema vesiculosum* is characterized by the appearance of numerous pinhead-sized or smaller transparent vesicles containing pure serum. The lesions have a tendency to rupture soon after their development, discharging their contents upon the surface. The discharge may continue in the form of a slow oozing, which is a decided feature of the disease and is regarded as the acme of the process. A continuance of this condition forms the variety described as *eczema rubrum or madidans.* The vesicles may remain intact where the epidermis is thick, as upon the palms of the hands and about the fingers, but being pricked with a needle give exit to a small quantity of serum. About the fingers and toes they sometimes coalesce to form bleb-like lesions, and rupturing, discharge their contents. The exudation of serum, instead of continuing, may dry on the surface, forming crusts which sooner or later fall off and reveal a desquamating surface. Burning and itching are decided symptoms.

*Eczema pustulosum* may develop from the variety just described, the contents of the vesicles becoming purulent, or it may consist of a primary eruption of pustules. Pustular eczema is met with most frequently on the scalp of children. The pustules rupture and their contents dry upon the surface, forming greenish or yellowish crusts.

*Eczema rubrum, or madidans*, develops from the foregoing varieties, especially the vesicular, and is that condition in which the cutaneous surface presents a dark red, tense, congested appearance and an oozing of serum. The surface is moist with the discharge, which varies in quantity, it being considerable at first and gradually growing less, finally drying and forming thin grayish or yellowish crusts. The discharge is serum, pure or mixed with blood or pus. It is tenacious or gummy and contains albumen. Eczema rubrum is most commonly encountered on the face and ears of children and about the genitalia and lower extremities.

*Eczema squamosum* represents the final stage of the disease and follows each of the preceding varieties. It is the stage through which the disease must pass before a normal condition is resumed. There is present more or less thickening of the skin, and the surface of the lesions are usually pale and covered with variously sized scales. This desquamation may continue indefinitely, and on the hands, where it is of common occurrence, it forms an obstinate affection.

*Eczema fissum* is that variety in which the eczematous process is accompanied by fissures or clefts in the skin. It occurs more as a result of the preceding varieties when the latter affect certain localities where the integument is liable to be put upon the stretch, as about the joints, especially of the hands, also about the mouth, ears and anus. When the fissures are deeply seated they are more or less painful, and there may be some serous or bloody exudation which, on drying, forms crusts.

The conditions described in these different varieties mainly constitute the symptoms of eczema. They may be present singly or in varied combinations presenting different stages of the development (progression and retrogression) of the disease. A part or the entire cutaneous surface may be affected. As a rule, however, it attacks only limited portions, and exhibits a predilection for particular localities, as the head, the hands and feet, lower extremities and genitals. It has a tendency to spread by continuity, also reflexly. An illustration of the latter mode is seen in those cases in which acute eczema of the hands or scrotum is followed by a sudden outbreak of the disease on the face or ears.

The disease runs an acute or chronic course. Acute eczema is usually preceded by constitutional disturbance, such as restlessness, fever, general malaise, also gastric symptoms. It runs its course, as a rule, in two to eight weeks, the skin recovering completely and revealing no sign of the inflammation to which it had been subjected. When it involves the whole surface (universal eczema), decided febrile symptoms are present. The eruption appears in some locality and is soon followed by the appearance of lesions in other parts which gradually increase in size, coalesce and form large patches. Different varieties are usually present in such a case. In one part papular lesions may be seen, in another vesicular, in another the surface may be oozing; again there may be large desquamating areas. It requires several months for such developments, and when healing occurs there are usually residua, such as fissures about the ears and flexures of the joints.

Acute eczema is generally confined to some particular region. It is common about the head (scalp, face, and especially the ears). On the face it causes swelling, and the eyelids may become so œdematous that they cannot be opened. The oozing of serum is profuse, especially about the ears. This is followed by crusting, and on the scalp, if neglected, the hair becomes matted. Affecting the penis and scrotum it causes considerable œdema and abundant oozing of serum. In the flexures of the thighs and other localities where the surfaces of the skin are in apposition, the epidermis may become macerated through friction and confined perspiration, and the cutaneous tissues become the seat of a diffuse redness (erythema intertrigo) which, if allowed to continue, is followed by and exudation of serum, producing a condition known as *eczema intertrigo*.

Acute eczema of the hands and feet is usually of the vesicular variety, and affecting the fingers and toes, where there is much swelling, it may interfere with mobility. Affecting the dorsum of the hands and feet there is usually some tumefaction. On the palms of the hands the process may continue in the squamous form. In all these localities the process may relapse or continue in a subacute manner, being then called chronic.

Chronic eczema differs from acute eczema only in its persistence and its results, being characterized by its quiescent condition. It undergoes little change from time to time, but is accompanied by paroxysms of intense itching, which compel the patient to scratch the lesions. This is a constant source of irritation that occasionally renders the process acute. Chronic eczema may also from other causes be converted into the acute form. The results of chronic eczema are thickening of the tissues, due to an increase of connective tissue, and brownish discoloration of the skin, due to a deposition of pigment. Long continued on the lower extremities it not infrequently leads to ulceration.

Chronic eczema, being a continuation of the acute form, is therefore encountered in the same localities as the latter. On the scalp it occurs in the pustular variety most frequently in children, also as chronic eczema rubrum or squamosum, the former giving rise to thick, adherent crusts, the latter to dry scales. The affection may extend on to the forehead and, as a rule, involves some part or all of the auricles. When confined to the occipital region it is, almost without exception, associated with pediculosis. The hair may fall out, but generally returns. The cervical glands may be enlarged. Affecting the ears, the disease is accompanied by the development of fissures. On the face it may occur in certain limited parts—on the upper eyelids, causing thickening, with constant scaling—about the alæ of the nose and upper lip, extending on to the mucous membrane of the nostril, due to irritating nasal discharges—on the hairy portions of the face in the form of chronic eczema rubrum or squamosum. These limited lesions are proverbially obstinate to treatment. On the mammary gland of the female the affection is usually confined to the region about the nipple. The skin is more or less swollen and the surface is moist with oozing serum or covered at times with crusts. The nipple may become the seat of painful fissures, and where the disease is allowed to develop it gradually becomes retracted and disappears.

Paget described a form of eczema, affecting the region about the nipple, which may be followed by cancer of the mammary gland. Whether this form is the result of a long-continued, neglected chronic eczema, or is a malignant process from the beginning, is not yet determined. It is probable that the cancerous change is here induced from the chronic inflammatory process in individuals predisposed, just as it is caused in other parts by continued surface irritation. The fact remains, however, that mammary cancer follows chronic cutaneous disease about the nipple. A microscopical examination of the specimens from two such cases made by Duhring and myself (Am. Journal, Med. So., July, 1884) indicated an intimate relation between the cutaneous disease and the malignant disease of the gland. The process, as it existed in the skin, was no longer of an eczematous nature; whether it ever had been was not evident. The epithelial structure of the skin was seen to be in a state of proliferation and the papillæ of the corium were found compressed and in many places entirely wanting. Extending along the lactiferous tubes, the process involved the glandular structure and the well-known characteristics of scirrhus cancer were plainly visible. Eczematous affections of the regions around the nipple should, therefore, command prompt attention and judicious treatment.

Eczema of the genitals is one of the most distressing as well as obstinate forms of disease. In the male it occurs on the penis and scrotum, ordinarily as eczema rubrum, discharging profusely and accompanied by more or less œdema and thickening. The decomposition of the discharge in neglected cases gives rise to a disagreeable odor. The exudation ceases at times and desquamation takes place, but fresh hyperemiæ may occur, followed by vesiculation and oozing, which may extend on the thighs even to the knees. The itching is tormenting, being worse at night, often keeping the patient awake. The same form attacks the labia majora and minora in the female, causing œdema of the parts. The disease may invade the perineum and anus, and in the latter place may be accompanied by the development of inflammation of the rectum and prolapse, also fissures that give rise to intense pain on defecation.

On the arms and legs chronic eczema occurs generally symmetrically and mostly on the flexor surfaces. Below the knees it is mostly in the form of chronic eczema rubrum and it is often associated with a varicose condition of the veins. The skin is thickened, intensely congested, mostly of a dark red, violaceous hue, discharging serum or covered here and there with dirty, yellowish or grayish crusts, or in a state of desquamation. Itching is always a symptom. The process on healing leaves a brownish discoloration of the part.

Chronic eczema of the hands and feet may be presented in any of the varieties, most commonly, however, as vesicular and squamous. In the vesicular variety the lesions are prominent on account of the thickness of the epidermis. About the fingers and toes painful fissures extending to the corium may be noted.

